# A Study of Blood Fatty Acids Profile in Hyperlipidemic and Normolipidemic Subjects in Association with Common *PNPLA3* and *ABCB1* Polymorphisms

**DOI:** 10.3390/metabo11020090

**Published:** 2021-02-04

**Authors:** Thomai Mouskeftara, Antonis Goulas, Despoina Ioannidou, Charikleia Ntenti, Dimitris Agapakis, Andreana Assimopoulou, Helen Gika

**Affiliations:** 1 Laboratory of Forensic Medicine and Toxicology, School of Medicine, Aristotle University of Thessaloniki, 54124 Thessaloniki, Greece; mthomaiv@auth.gr; 2 Biomic AUTh, Center for Interdisciplinary Research and Innovation (CIRI-AUTH), 57001 Thessaloniki, Greece; 3 Laboratory of Pharmacology, School of Medicine, Aristotle University of Thessaloniki, 54124 Thessaloniki, Greece; agoulas@auth.gr (A.G.); depioannidou@gmail.com (D.I.); chntenti@gmail.com (C.N.); 4 Department of Internal Medicine, AHEPA Hospital, School of Medicine, Aristotle University of Thessaloniki, 54124 Thessaloniki, Greece; dimagap@yahoo.gr; 5 Natural Products Research Center of Excellence (NatPro-AUTH), Center for Interdisciplinary Research and Innovation (CIRI-AUTH), 57001 Thessaloniki, Greece; adreana@cheng.auth.gr; 6 Laboratory of Organic Chemistry, School of Chemical Engineering, Aristotle University of Thessaloniki, 54124 Thessaloniki, Greece

**Keywords:** PNPLA3, ABCB1, GC-FID, fatty acids profile, FAME

## Abstract

Adiponutrin (patatin-like phospholipase domain-containing 3; PNPLA3), encoded in humans by the *PNPLA3* gene, is a protein associated with lipid droplet and endoplasmic reticulum membranes, where it is apparently involved in fatty acid redistribution between triglycerides and phospholipids. A common polymorphism of *PNPLA3* (I148M, rs738409), linked to increased *PNPLA3* presence on lipid droplets, is a strong genetic determinant of non-alcoholic fatty liver disease (NAFLD) and of its progression. P-glycoprotein (Pgp, MDR1—multidrug resistance protein 1, ABCB1—ATP-binding cassette sub-family B member 1), encoded by the *ABCB1* gene, is another membrane protein implicated in lipid homeostasis and steatosis. In the past, common *ABCB1* polymorphisms have been associated with the distribution of serum lipids but not with fatty acids (FA) profiles. Similarly, data on the effect of *PNPLA3* I148M polymorphism on blood FAs are scarce. In this study, a gas chromatography-flame ionization detection (GC-FID) method was optimized, allowing us to analyze twenty FAs (C14: 0, C15: 0, C15: 1, C16: 0, C16: 1, C17: 0, C17: 1, C18: 0, C18: 1cis, C18: 2cis, C20: 0, C20: 1n9, C20: 2, C20: 3n6, C20: 4n6, C20: 5, C23: 0, C24: 0, C24: 1 and C22: 6) in whole blood, based on the indirect determination of the fatty acids methyl esters (FAMES), in 62 hyperlipidemic patients and 42 normolipidemic controls. FA concentrations were then compared between the different genotypes of the rs738409 and rs2032582 (*ABCB1* G2677T) polymorphisms, within and between the hyperlipidemic and normolipidemic groups. The rs738409 polymorphism appears to exert a significant effect on the distribution of blood fatty acids, in a lipidemic and fatty acid saturation state-depending manner. The effect of rs2032582 was less pronounced, but the polymorphism did appear to affect the relative distribution of blood fatty acids between hyperlipidemic patients and normolipidemic controls.

## 1. Introduction

Patatin-like phospholipase domain-containing 3 (PNPLA3), also known as adiponutrin (ADPN) or calcium independent phospholipase A2ε (iPLA2ε), is a protein tightly associated with lipid droplet and endoplasmic reticulum membranes; it displays in vitro triglyceride (TG) hydrolase as well as retinyl hydrolase activities and a modest acyltransferase activity was also reported in some studies [1]. *PNPLA3* is highly expressed in hepatocytes and in hepatic stellate cells where it is believed to function as a major regulator of lipid droplet content, by promoting TG and phospholipid (PL) remodeling [2]. A common polymorphism in the human *PNPLA3* gene (rs738409), originating in a cytosine to guanine substitution which directs an isoleucine to methionine change at position 148 (I148M) of the *PNPLA3* primary structure, is a strong genetic determinant of non-alcoholic fatty liver disease (NAFLD) and of its progression; homozygotes for the minor allele (G) present with a 10-fold risk of developing hepatocellular carcinoma compared to the other genotypes [3]. The I148M change confers to the protein resistance to proteasomal degradation [4] and reduction of the hydrolase activities [5], leading to accumulation of mainly monounsaturated fatty acids in the lipid droplet and decreased secretion of very low density lipoprotein (VLDL) from the hepatocyte; at the same time the transfer of polyunsaturated fatty acids (PUFAs) from TGs to PLs appears to be enhanced in presence of the mutant protein in genetically modified mice [2] albeit, paradoxically, the opposite was observed in the human liver [6].

While the effect of *PNPLA3* I148M on hepatic lipid homeostasis and NAFLD is being thoroughly studied, its association with serum lipids is much less examined; a small number of reports have suggested association of the *PNPLA3* 148M allele with decreased serum TG levels, in individuals with impaired glucose regulation [7], in the obese [8], and in patients with gallstones [9]. Along the same lines, Hyysalo et al. [10] have reported an association of *PNPLA3* 148M with lower serum TG concentrations in a population-based study. Information concerning the relative prevalence of individual fatty acids (FAs) in 148M carriers and non-carriers is even more limited; however, recently Luukkonen et al. [6] reported that the 148M allele increases retention of PUFAs in the livers of obese subjects, resulting, in turn, in PUFA-deficient VLDL-TGs in the circulation. As FA profiles in the circulation have been linked to metabolically unhealthy phenotypes [11,12], examining the association of the *PNPLA3* I148M with distinct blood FA profiles in separate groups of patients and controls can add to our knowledge concerning the clinical significance of this polymorphism.

The P-glycoprotein (Pgp, MDR1, ABCB1) is another membrane protein implicated in lipid homeostasis; in addition to its well defined role as an efflux pump, Pgp has been linked to intracellular cholesterol trafficking [13,14], transintestinal cholesterol excretion [15], and translocation of phospholipids and sphingolipids [16]. Moreover, diet restriction was shown to induce a very strong up-regulation of the ABCB1a gene (one of the two mice orthologs of the human *ABCB1* gene) in mice [17] and, more importantly, Pgp knock-out mice developed obesity, hepatic steatosis and increased liver TGs [18]. Common polymorphisms of the *ABCB1* gene have been associated in the past with serum total cholesterol and/or lipoprotein cholesterol levels [19], but, as in the case of *PNPLA3* gene, the effect of such polymorphisms on individual FA concentrations in human serum remains largely unexplored.

Determination of individual FAs and exploration of their profiles has been assisted greatly by the development of lipid analysis methodologies. Here, a FA profiling method was applied to provide an insight in the composition of blood samples from a subset of previously characterized hyperlipidemic patients and normolipidemic controls [19]. The applied method provides reliable quantitative results by the indirect determination of FAs’ more volatile methyl ester derivatives (FA methyl esters, FAMEs) by gas chromatography and flame ionization detection (GC-FID). A two-stage sample preparation protocol, including extraction of the lipid fraction by the Folch method and esterification of the total fatty acid content by methanol in acidic conditions was applied [20]. The distributions of total as well as individual blood FAs were then compared between carriers of different genotypes of the *PNPLA3* I148M (rs738409) and *ABCB1* 2677G>T (rs2032582) polymorphisms in a group of previously characterized hyperlipidemic patients [16] with the aim to explore associations between the circulating FAs and genotypes which could be of clinical interest. As such associations may be affected by the degree of metabolic burden [21], a group of similarly characterized normolipidemic patients [16] was also included in our study.

## 2. Results

### 2.1. Blood FA Profiling

#### 2.1.1. Analytical Performance of the Method

**Figure 1:**
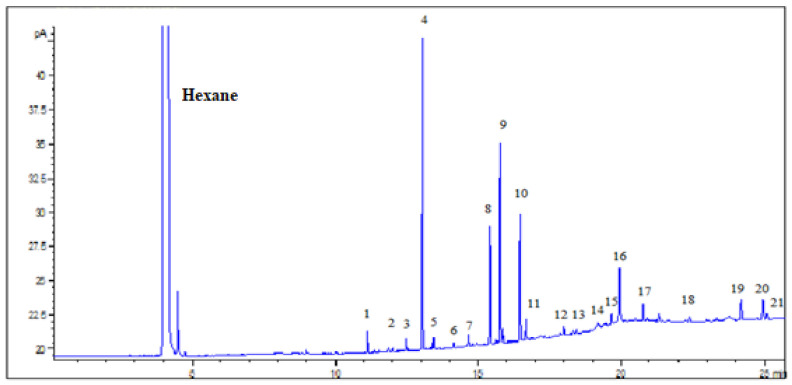
Chromatogram of whole blood sample after pretreatment and esterification of FAs with CH_3_OH / HCl. Peaks 1–21: C14:0, C15:0, C15:1, C16:0, C16:1, C17:0, C17:1, C18:0, C18:1cis, C18:2cis, C19:0 (injection standard), C20:0, C20:1n9, C20:2, C20:3n6, C20:4n6, C20:5, C23:0, C24:0, C24:1, C22:6, respectively.

The developed method was able to detect twenty FAs in the analyzed samples. A representative chromatogram is given in Figure 1 where the peaks of the FA methyl esters in a sample can be seen. Regarding the validity of the analytical results, method’s evaluation parameters were examined. Method’s linearity was satisfactory for the concentration range, which was studied, providing a correlation coefficient R^2^ > 0.990 for all FAMEs and satisfactory LODs (0.002–3.79 mmol/L) and LOQs (0.02 mmol/L–12.64 mmol/L). Analytical figures of merit are summarized in Table 1.

Intra-assay and inter-assay accuracy ranged from 72.49% (C17: 0) to 109.30% (C20: 4 n6), and from 72.54% (C14:0 cis) to 106.16% (C20: 4 n6), respectively. Precision in RSD% values ranged from 0.22% (C20: 4 n6) to 14.06% (C20: 0) within a batch and from 0.59% (C20: 4 n6) to 11.12% (C20: 0) between batches over a period of one week. Stability of the analytes in the freezer at −20 °C was satisfactory basically up to 3 weeks. The results expressed as recovery % are ranging from 79.40% (C15: 1) to 109.05% (C22: 6) 24 h after freezing, from 72.39% (C16: 0) to 105.95% (C22: 6) 48 h after, from 70.56% (C15: 1) to 98.59% (C23: 0) 3 weeks after and 69.97% (C16: 0) to 96.80% (C17: 0) 3 months after freezing.

#### 2.1.2. FA Concentrations

Determination of FAs was performed in blood samples of 62 hyperlipidemic patients and 42 normolipidemic controls, constituting representative subgroups in terms of *ABCB1* G2677T [19] and *PNPLA3* I148M genotype distribution- of the original groups. The original selection was based on known *ABCB1* G2677T genotypes; *PNPLA3* I148M genotyping failed to produce unequivocal results for one hyperlipidemic patient and two normolipidemic controls. Patients with extreme triglyceride values (>5.5 mmol/L) were excluded. A summary of demographic, biochemical and genetic characteristics of the sample subset is shown in Table 2.

Mean average of individual FA concentrations in the two groups is shown in Table 3. (Detailed data on individual FA concentrations for every analyzed sample is provided in supplementary information Table S1). As seen, the most abundant FAs among normolipidemics, in descending order, were C16:0 > C18:1 > C18:0 > C20:4 > C18:2 > C20:5, and in hyperlipidemics, C18:1 > C16:0 > C18:0 > C18:2 > C20:4 > C20:5. While the total FA concentration was significantly increased in hyperlipidemics compared to normolipidemics, this increase was not applied to every individual FA detected. More specifically, statistically significant increases were observed with respect to C14:0, C15:0, C16:0, C16:1, C17:0, C18:1, C18:2, and C20:3n6. Nominally significant decreases were also observed, for C20:4n6, C24:1 and C22:6, none of which survived Bonferroni correction, however.

### 2.2. Association of FA Concentrations with *ABCB1* G2677T and *PNPLA3* I148M Genotypes

#### 2.2.1. Total Blood FAs

To probe the association of total blood FA concentration with the two polymorphisms, in the two groups (Table 4, Figure S1), an Analysis of covariance (ANCOVA) type of analysis was used which allowed us to use body mass index (BMI) and glycated hemoglobin (HbA1c) as covariates, since these parameters displayed nominal associations with *PNPLA3* I148M (*p* = 0.036 and 0.021, for BMI and HbA1c, respectively) in preliminary analyses. Our findings indicate that (i) the *PNPLA3* I148M polymorphism is associated with the total blood FA concentration in a group-dependent fashion, with GG (MM) genotypes displaying clearly higher concentrations compared to the *PNPLA3* 148C (I) carriers, but only in the hyperlipidemic group, (ii) a strong trend for allele-dosage effect of the *ABCB1* G2677T polymorphism is apparent, similarly limited to the hyperlipidemic group, and (iii) significant difference in total blood FA concentration between the two groups is limited to the genotypes that are homozygous for the minor allele of one or the other polymorphism (*PNPLA3* 148MM and *ABCB1* 2677TT, respectively).

#### 2.2.2. Individual Blood FAs

**Figure 2:**
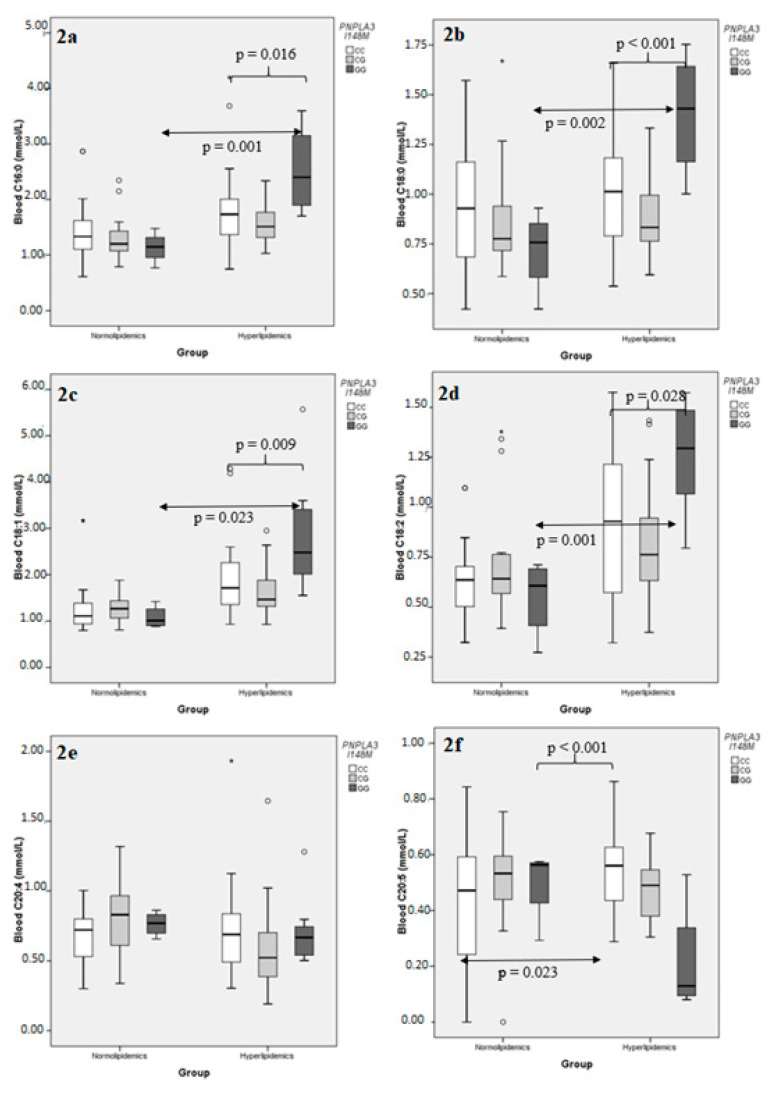
Comparison of the distributions of individual blood fatty acid concentrations following stratification according to *PNPLA3* I148M genotypes, in normolipidemic controls and hyperlipidemic patients. (**a**) C16:0; (**b**) C18:0; (**c**) C18:1; (**d**) C18:2; (**e**) C20:4; (**f**) C20:5. A small circle indicates an outlier, defined by SPSS as extending within 1.5 and 3 lengths of the interquartile range from the edge of the corresponding box. An asterisk indicates an extreme value, defined by SPSS as extending more than 3 lengths of the interquartile range from the edge of the box.

**Figure 3:**
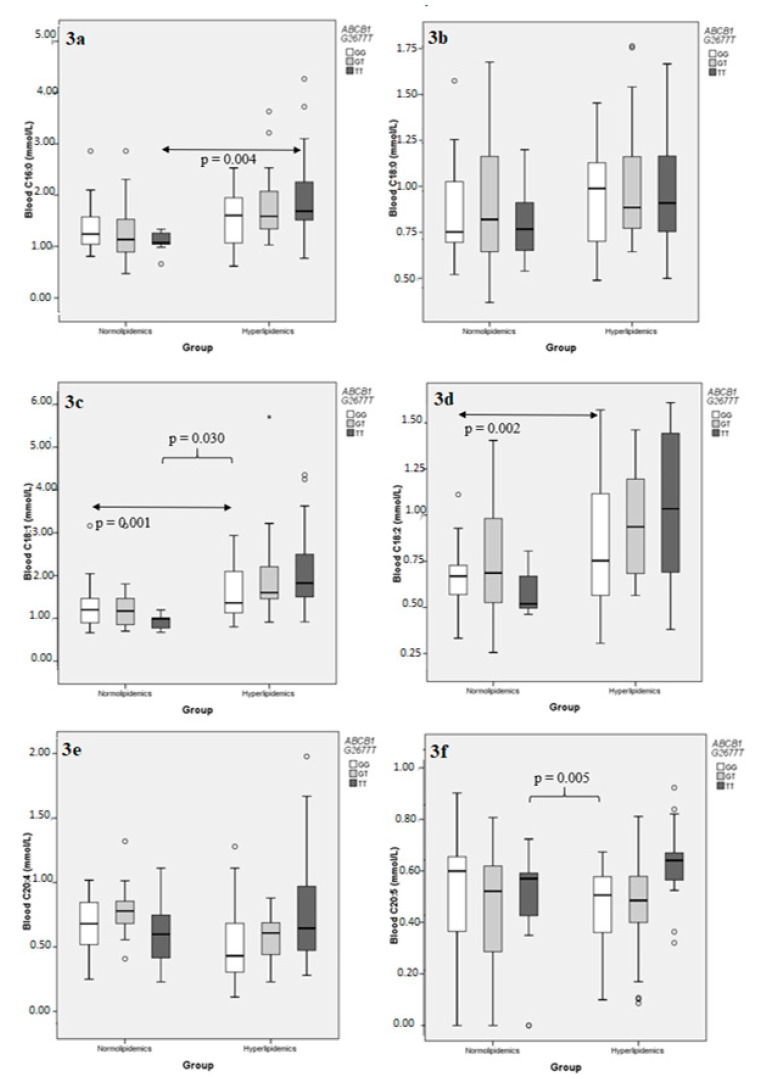
Comparison of the distributions of individual blood fatty acid concentrations following stratification according to *ABCB1* G2677T genotypes, in normolipidemic controls and hyperlipidemic patients. (**a**) C16:0; (**b**) C18:0; (**c**) C18:1; (**d**) C18:2; (**e**) C20:4; (**f**) C20:5. A small circle indicates an outlier, defined by SPSS as extending within 1.5 and 3 lengths of the interquartile range from the edge of the corresponding box. An asterisk indicates an extreme value, defined by SPSS as extending more than 3 lengths of the interquartile range from the edge of the box.

The same type of analysis was repeated for individual FAs, taken into consideration the six most abundant ones, namely C16:0, C18:0, C18:1, C18:2, C20:4, and C20:5 (Tables S2–S7). Examination of the effect of the *PNPLA3* I148M polymorphism on the blood concentration of the aforementioned FAs the hyperlipidemic group revealed that, while the distributions of C16:0, C18:0, C18:1, and C18:2 appeared to follow the general pattern described for total FAs, the polyunsaturated C20:4 and C20:5 behaved differently; *PNPLA3* I148M did not affect C20:4 concentration in either group, while its effect on C20:5 values was opposite compared to the rest, in that *PNPLA3* 148MM genotypes had significantly lower, rather than higher, C20:5 concentrations compared to the *PNPLA3* 148I carriers (Figure 2). A multiple sequential regression analysis, using *PNPLA3* I148M as a dichotomous independent variable (148I carriers vs. 148MM), BMI and HbA1c as continuous independent variables, and the same FA concentrations as separate dependent variables, confirmed the above result and further established that neither BMI nor HbA1c had any significant effect added to that of *PNPLA3* I148M, as the adjusted R^2^ (a measure of the independent variable’s explaining power on the variation) was not significantly altered upon addition of BMI first and then HbA1c to the model for any FA examined, with the single possible exception of C18:1, where a nominal effect of the addition of HbA1c as an independent variable was noted (Table 5). On the other hand, the effect of *ABCB1* G2677T polymorphism on the same individual FAs reproduced the trend observed for the entire sum, with statistical significance achieved only for C20:5, but without the directional variation observed with *PNPLA3* I148M (Figure 3; Tables S2–S7). As in the case with total FA concentrations, no significance effect of either genotype was detected in the normolipidemic group.

When genotype-stratified individual FA concentrations were compared between the normolipidemic and the hyperlipidemic groups, statistically significant differences (or trends thereof) were largely confined to the *PNPLA3* 148MM or *ABCB1* 2677TT, as was the case with total FAs, with the exception of C20:4 where only *ABCB1* 2677GT heterozygotes differed significantly in their respective blood concentrations following Bonferroni correction, and C18:0 where stratification according to the *ABCB1* 2677G>T polymorphism produced no statistically significant effect (Tables S2–S7).

## 3. Discussion

One major finding of this study was that the *PNPLA3* I148M (C > G; rs738409) polymorphism affects the distribution of blood fatty acids in a lipidemic and fatty acid saturation state-depending manner. More specifically, hyperlipidemic carriers of the *PNPLA3* 148MM (GG) genotype displayed apparently higher blood concentrations of palmitic, stearic, oleic and linoleic acid, similar concentrations of arachidonic acid and lower concentrations of eicosapentaenoic acid, compared to carriers of the major C allele (*PNPLA3* 148II or *PNPLA3* 148IM). This observation is in line to some of the results published recently by Luukkonen et al. [6], according to which the ratio of mean absolute concentrations of plasma very low density lipoprotein-associated triglycerides (VLDL-TGs) in carriers of the *PNPLA3* 148MM genotype, as compared to carriers of the *PNPLA3* 148II genotype, is inversely related to the degree of unsaturation of their corresponding FAs. The authors attributed that observation to the increased retention of PUFAs in the liver of *PNPLA3* 148MM carriers, compared to *PNPLA3* 148II carriers [6,22] As most fatty acids in blood are contained in VLDL-TGs, our results paralleled those described above. However, there are some recorded differences: Participants of the Luukkonen study [6] were mostly obese but normolipidemic whereas the subjects in our study displaying the effect of *PNPLA3* I148M on blood fatty acids were mostly overweight, but not obese, and hyperlipidemic. At this point, we cannot offer a detailed explanation as to why this effect was not observed in our normolipidemic group, other than speculating that this could be related to the TG burden of the liver. Unfortunately, the retrospective nature of our study and the lack of relevant markers in the patient’s clinical files precluded us from obtaining information concerning liver fatty acid data and/or the NAFLD status of the participants. However, the fact that neither BMI nor HbA1c proved to be significant determinants of within group FA variation in our study, points against an independent effect of BMI or type 2 diabetes [10]. On the other hand, the inclusion in our study of heterozygotes with respect to the adiponutrin polymorphism (*PNPLA3* 148IM) allow us to agree with the designation of the *PNPLA3* 148M as a “loss of function” allele, since the heterozygotes displayed blood FA profiles similar to those of the *PNPLA3* 148II (major allele) homozygotes in our study.

Another interesting observation with respect to *PNPLA3* I148M polymorphism, was that the overall increase in blood FAs which was recorded in hyperlipidemics was limited to the carriers of the MM genotype, providing additional support for the significance of adiponutrin in FA homeostasis, which goes apparently beyond its proposed role in lipid remodeling by simply transferring FAs from TGs to PLs [1]. Our findings, and those of Luukkonen et al. [6], appear to weaken the argument that the overabundance of *PNPLA3* in lipid droplets simply reduces the overall transfer of FAs to nascent VLDL particles and, thereby, their release from the hepatocyte [1]. Rather, the saturation specific increase of blood FAs in *PNPLA3* 148MM hyperlipidemic compared to *PNPLA3* 148MM normolipidemic subjects is more consistent with the retention of C20:5-enriched TGs in the liver, at the expense of the less unsaturated and saturated FAs which end up preferentially populating circulating lipoproteins, as argued before [6]. Any reduction in VLDL secretion could in turn be explained by the aberrant lipidation of apoB which was proposed to increase the rate of its proteosomal-independent post-translational degradation [23]. This retention of C20:5 could, in addition, protect *PNPLA3* 148MM carriers from insulin resistance, as saturated fat appears to be more harmful for the liver [22]. Indeed, in our original cohort, none of the *PNPLA3* 148MM genotypes presented with a HbA1c value indicative of T2D (Table S8 and unpublished results). However, whether this confers protection from atheromatosis independently from insulin-resistance, is another question; it should be noted that a metabolically healthy profile has been generally associated with higher rather than lower levels of PUFAs in blood [11]. On the other hand, while this manuscript was in preparation, a study was published reporting that the presence of *PNPLA3* 148M allele is associated with lower numbers of circulating LDL and VLDL particles and, therefore, an antiatherogenic effect, but largely confined to morbidly obese and insulin-resistant individuals [21].

The effect of the *ABCB1* 2677G>T (rs2032582) polymorphism on FA distribution observed in this study may not be as clear-cut as that of *PNPLA3* I148M described above but does constitute a novel finding. While a trend of allele dosage effect was apparent, limited—again—in the hyperlipidemic group, it did not reach statistical significance overall nor did it hint at an association with FA saturation as was the case with the *PNPLA3* polymorphism. Since in humans common *ABCB1* polymorphisms including rs2032582 and its tightly linked rs1045642 have in the past been associated mainly with blood cholesterol (total, LDL-, or HDL-associated) rather than triglycerides [19], the fact that the overall difference in blood fatty acids between the two groups appears to be *ABCB1* 2677G>T allele-specific, and is more pronounced for C20:5, suggest a possible effect on PLs and/or sphingolipids (SLs). Pgp is indeed known to translocate PLs and SLs, among other things, and their transport efficacy may well be related to structural elements of their FA content, such as degree of saturation and hydrocarbon chain length [16]. As Pgp is present in the membranes of the organelles involved in VLDL synthesis, intracellular trafficking and secretion [24], a selectivity of that sort could affect the pool of PLs and SLs available for export in the circulation and, thus, influence the constitution of those retained in the liver. Pgp could thus be linked to lipid remodeling in the liver; mice in which both mdr1 genes (as opposed to humans who only have one, mice carry two such highly homologous genes) had been knocked out (mdr1ab-/-) displayed a dramatic retention of TGs in their livers and developed liver steatosis and obesity following a 25- or 35-week regimen of either standard or high-fat diet, despite having a genetic background otherwise resistant to diet-induced obesity [18]. In a separate experiment, C57BL/6 mice fed a restricted diet for three weeks displayed an impressive increase in hepatic mdr1a expression [17]. Also, the knocking out of heat shock factor-1, which reversed obese phenotype and a propensity for formation of atherosclerotic lesions in mice depleted of LDL receptors, was associated with the up-regulation of P-gp expression in the liver [25], and *ABCB1* was one of the target genes recently identified in a search for compounds active against liver fibrosis with an integrated bioinformatics analysis [26].

This study has many limitations: A retrospective design with its use of stored rather than fresh blood samples which may have affected lipid stability in a selective manner, the lack of imaging or biochemical data pertaining to NAFLD status and insulin resistance, and the small number of *PNPLA3* 148MM genotypes in the normolipidemic group are only but a few. Nevertheless, we are confident that it did provide reliable data consistent with the hypothesis that the *PNPLA3* I148M polymorphism affects the distribution of FAs between liver and general circulation in a saturation-dependent manner and attests to the importance of using lipidomics for fine analysis of FA distribution in various groups. It also serves as a reminder that Pgp most likely plays an underappreciated role in lipid homeostasis which warrants additional study.

## 4. Materials and Methods

### 4.1. Chemicals and Reagents

All solvents used were of analytical or LC-MS grade. Methanol LC-MS grade was purchased from CHEM-LAB NV (Zedelgem, Belgium), *n*-Hexane HPLC grade, was purchased from LiChrosolv (Merck, Darmstadt, Germany), Trichloromethane (CHCl_3_) Pro-analysis and Hydrochloric acid 37%, were purchased from Panreac AppliChem (Darmstadt, Germany). The solution 8% HCl/CH_3_OH was freshly prepared by diluting HCl 37% in LC-MS grade methanol. Ultra-pure water was prepared using a Milli-Q system (Millipore, Billerica, MA, USA). Methyl-nonadecanoate (Purity ≥ 98%) used as injection standard was supplied from Sigma-Aldrich (Darmstadt, Germany). The mixture of 37 fatty acid methyl esters (C4:0–C24:1) was supplied by Supelco (Darmstadt, Germany). Working solutions of FAMEs were prepared in hexane at 3 groups of concentration. Group 1 (20, 50, 100, 200 µg/mL), group 2 (40, 100, 200, 400 µg/mL), and group 3 (60, 150, 300, 600 µg/mL). For calibration 50 μL of a blood pooled sample was fortified at concentration levels of 2, 5, 10, 20 µg/mL (group 1), 4, 10, 20, 40 µg/mL (group 2) and 6, 15, 30, 60 µg/mL (group 3) with the addition of 5 μL of the proper working solution. Quality control samples were prepared by fortifying a pooled blood sample at concentrations of 2, 10, 20 µg/mL (group 1), 4, 20, 40 µg/mL (group 2) and 6, 30, 60 µg/mL (group 3).

### 4.2. Biological Samples

Information pertaining to the original groups of hyperlipidemic patients and normolipidemic controls can be found in [19] and in [27]. The participants were all Greek nationals and residents of Northern Greece. Selection of the samples included in this study was based on previously recorded *ABCB1* genotypes, to ensure adequate representation of all three (GG, GT, TT), in both hyperlipidemic patients and controls. Genotyping of *PNPLA3* I148M was done concurrently with FA profiling. All blood samples had been stored at 80 °C till processing. The study was under the approval of the Bioethics Committee of the Medical School of the Aristotle University of Thessaloniki protocol approval number 1/18.12.2013.

### 4.3. Genotyping

Peripheral blood collection for the determination of lipid parameters and genomic DNA isolation was performed following patient informed consent. Genomic DNA was isolated from venous blood using a commercially available kit (Ron’s Blood DNA minikit, Bioron GmbH, Ludwigshaften, Germany). The rs738409 genotypes were determined according to a previously established PCR-RFLP method [28]. Briefly, a 157 bp amplicon harboring the polymorphic site was produced by using TACCACGCCTCTGAAGGAAG and CCCTGCTCACTTGGAGAAAG as the forward and the reverse primer, respectively. Amplification conditions were: 5 min at 95 °C, followed by 37 cycles of 30 s at 95 °C, 30 s at 58 °C, and 30 s at 72 °C, and a final extension step of 7 min at 72 °C. The PCR mixture was then treated overnight with FokI (SibEnzyme, Academtown, Siberia, Russia) at 37 °C; the enzyme digests in presence of the C allele (sense strand) to produce 99 and 58 bp fragments, but not in presence of the G allele (sense strand) to leave the 157 fragment uncut. Genotyping data for rs2032582were retrieved from work previously accomplished in our laboratory [19,27].

### 4.4. FA Profiling

#### 4.4.1. Sample Preparation Procedure

Lipid fraction was first extracted from whole blood samples by the Folch method [18]. An aliquot of 50 μL of blood was transferred in a 1.5 mL vial and 850 μL of chloroform-methanol, 2:1 (*v*/*v*) was added, followed by stirring for 20 min. One hundred and eighty microliters of water were then added to facilitate the separation of organic and aqueous phase and the extract was centrifuged at 4000× *g* for 5 min. The lower layer of chloroform containing the lipids was transferred to a 10 mL glass vial and the solvent was evaporated to dryness under a stream of N_2_. Transesterification was performed by the addition of 3 mL of methanol, 600 μL of 8% CH_3_OH-HCl solution in the dry residue and heat in gat 100 °C for 90 min. The extract was then left to reach room temperature and 2 mL of hexane were added and the vial was shaken at a rotary shaker for 30 min to extract FAMEs. Centrifugation followed at 1000× *g* for 5 min to receive the hexane layer. The hexane extract was dried under a stream of N_2_ and the dried residue was reconstituted with 0.5 mL of hexane. In this 7.5 μL of the external standard solution (nonadecanoic acid methyl ester, 5 mg/mL) were added and the sample was injected into the GC system.

#### 4.4.2. Gas Chromatographic Analysis

GC–FID analysis was performed by an Agilent Technologies 6890N Gas chromatograph system with flame ionization detector (Agilent Technologies, Wilmington, DE, USA). The separation of fatty acid methyl esters was accomplished using an Agilent J&W DB-23 column (60 m × 250 μm × 0.25 μm). Helium (purity 99.999%) was used as carrier gas at a constant flow rate of 1.3 mL/min. Injection of 1 μL sample volume was performed in split mode (20:1) at an inlet temperature of 250 °C. The oven temperature program was as follows: The initial temperature 50 °C was held for 1 min and then increased to 175 °C with a 25 °C/min rate. Then temperature then rose from 175 °C to 230 °C at a rate of 4 °C/min. The total run time was 26.8 min.

The detector temperature was set at 280 °C and the flame was maintained with 40 mL/min H_2_ and 450 mL/min air. Helium also used as a makeup gas at a flow of 30 mL/min. Identification of the FAs was based on the retention time.

#### 4.4.3. Method Validation

The method was evaluated according validation guidelines by FDA [29]. Calibration curves were constructed based on fortified pooled blood sample prepared by the whole set of samples. Five microliters of the standard solution containing the 37 methyl esters of FAs at 3 groups of concentration, group 1 (20, 50, 100, 200 µg/mL), group 2 (40, 100, 200, 400 µg/mL) and group 3 (60, 150, 300, 600 µg/mL) were placed in a 1.5 mL glass vial and evaporated to dryness under a mild stream of nitrogen. Then a 50 μL aliquot of blood sample was added and vortexed obtaining final concentrations of 2, 5, 10, 20 µg/mL (group 1), 4, 10, 20, 40 µg/mL (group 2) and 6, 15, 30, 60 µg/mL (group 3). A control sample was also used without fortification. After the sample preparation procedure described above in Section 4.4.1 was applied, the samples were analyzed and calibration curves were constructed based on the ratio of peak areas with the area of the injection standard for every analyte. LOD was calculated based on the mathematical equation LOD = 3 × SD (where SD is the standard deviation of the blank signal) for a 99% confidence interval, while LOQ limit was calculated based on the mathematical equation LOQ = 10 × SD (where SD is the standard deviation of the blank signal).

Precision and accuracy of the method were assessed by analyzing in replicates (*n* = 6) a control sample and 3 fortified blood samples at low (2, 4, 6 μg/mL), middle (10, 20, 30 μg/mL) and high (6, 30, 60 μg/mL) within the same analysis batch (intra assay) and in 3 different analytical batches over a period of a week (inter assay). The intra and inter assay accuracy and precision were expressed as recovery (R%) and as percentage relative standard deviation (RSD%).

The stability of the analytes was studied in spiked blood samples at 3 concentrations LQC (2, 4, 6 µg/mL), MQC (10, 20, 30 µg/mL) and HQC (20, 40, 60 µg/mL). Three replicate extracts for each concentration and one control sample were analyzed directly after their preparation and then were analyzed again 24 h, 48 h, 3 weeks, and 3 months after at −20 °C. Column overload was assessed by pure solvent (hexane) analysis after the injection of the highest concentration calibrator.

#### 4.4.4. Statistical Analysis

Continuous variables, including individual FA distributions, tended to deviate from normality; therefore, nonparametric tests (Mann–Whitney) were initially used for between-groups comparisons. Nevertheless, the need to include covariates (BMI, HbA1c) in the analyses of genotype-FA concentration associations, prompted us to use a univariate type III sum of squares statistics linear model (ANCOVA) for that purpose. For between-groups comparisons of genotype-stratified FA distributions, both Mann–Whitney and t-tests were used, with no differences in detected significance, the latter being reported for the sake of consistency. In the case of the *PNPLA3* I148M polymorphism, where initial data allowed stratification according to the recessive model for the minor allele (148M; G), a multiple regression analysis with fatty acid concentrations in the hyperlipidemic group as the dependent variable and *PNPLA3* I148M genotype (dichotomous: 148I carriers vs. 148MM), BMI and HbA1c as independent variables (sequential model) was used to confirm the results of the ANCOVA analysis. Categorical variables, including genotype distributions, were compared between the two groups with the *χ*^2^ test of independence. Possible deviations of the genotype distributions from the Hardy–Weinberg equilibrium were examined with the *χ*^2^ test of goodness-of-fit in each group. Bonferroni corrections were applied to the limit of statistical significance in multiple comparisons, leading to *p* = 0.0025 for between-group comparisons of all FAs; *p* = 0.0125 for within group ANCOVA analyses; *p* = 0.008 for between-group comparisons of individual genotype-stratified FAs

## Figures and Tables

**Table 1:** Retention times, linearity, coefficients of determination (R^2^), limits of detection (LOD), limits of quantification (LOQ) for all fatty acids (FAs) determined.

FA	Retention Time (min)	Linearity (mmol/L)	R^2^	LOD (mmol/L)	LOQ (mmo/L)
C14:0	11.151	0.014–0.415	0.991	0.001	0.004
C15:0	12.051	0.01–0.158	0.999	0.002	0.005
C15:1	12.516	0.01–0.449	0.993	0.002	0.007
C16:0	13.061	0.33–2.522	0.998	0.002	0.008
C16:1	13.461	0.01–0.825	0.992	0.002	0.005
C17:0	14.181	0.01–0.17	0.999	0.001	0.004
C17:1	14.621	0.03–0.521	0.996	0.007	0.024
C18:0	15.39	0.19–1.344	0.99	0.012	0.039
C18:1cis	15.772	0.32–2.95	0.991	0.034	0.112
C18:2cis	16.497	0.13–1.378	0.997	0.016	0.053
C20:0	18.013	0.007–0.071	0.998	0.002	0.005
C20:1n9	18.439	0.008–0.122	0.999	0.001	0.004
C20:2	19.2	0.006–0.092	0.997	0.001	0.003
C20:3n6	19.656	0.03–0.535	0.997	0.003	0.01
C20:4n6	19.957	0.29–0.993	0.997	0.027	0.089
C20:5	20.953	0.016–0.723	0.997	0.003	0.009
C23:0	22.4	0.01–0.189	0.999	0.003	0.009
C24:0	24.259	0.02–0.312	0.997	0.002	0.006
C24:1	24.987	0.03–0.533	0.998	0.003	0.009
C22:6	25.129	0.02–1.698	0.995	0.002	0.008

**Table 2:** Demographic characteristics and plasma lipid parameters of the study participants.

Demographic Characteristics and Blood Lipid Parameters	Group		*p* ^1^
	Normolipidemics	Hyperlipidemics	
*n*	42	62	
Age (years ± SD)	51.7 ± 14.38	55.0 ± 13.75	0.237
BMI (kg/m^2^ ± SD)	25.7 ± 2.91	27.3 ± 2.90	0.009
Sex (females, %)	54.5	65.1	0.313
Smoking (yes, %)	9.5	1.6	0.155
T2D ^2^ (yes, %)	16.7	36.5	0.030
HbA1c ^3^ (% ± SD)	NA ^4^	5.75 ± 0.927	–
TC ^5^ (mmol/L ± SD)	4.76 ± 0.689	6.53 ± 0.986	<0.001
TG ^6^ (mmol/L ± SD)	1.14 ± 0.440	2.40 ± 1.029	<0.001
*PNPLA3* I148M (*n*, %)			
CC (II)	21 (52.5)	26 (42.6)	0.616
CG (IM)	15 (37.5)	27 (44.3)	
GG (MM)	4 (10.0)	8 (13.1)	
Deviation from Hardy–Weinberg equilibrium, *p* ^7^	0.864	0.842	
*ABCB1* G2677T (*n*, %)			
GG	15 (35.7)	26 (41.9)	0.807
GT	16 (38.1)	22 (35.5)	
TT	11 (26.2)	14 (22.6)	
Deviation from Hardy–Weinberg equilibrium, *p* ^7^	0.327	0.115	

^1^ Continuous variables: Mann–Whitney test; categorical variables: *χ*^2^ test of independence; ^2^ type 2 diabetes at presentation; ^3^ glycated hemoglobin; ^4^ not available; ^5^ total cholesterol; ^6^ triglycerides; ^7^ *χ*^2^ test of goodness-of-fit.

**Table 3:** Fasting blood concentrations of FAs in hyperlipidemic patients and normolipidemic controls in mmol/L.

FA (mmol/L ± SD)	Group		*p* ^1^
	Normolipidemics	Hyperlipidemics	
C14:0	0.16 ± 0.079	0.25 ± 0.084	<0.001
C15:0	0.06 ± 0.021	0.08 ± 0.034	0.002
C15:1	0.29 ± 0.092	0.27 ± 0.087	0.468
C16:0	1.37 ± 0.487	1.81 ± 0.678	<0.001
C16:1	0.27 ± 0.209	0.45 ± 0.329	<0.001
C17:0	0.08 ± 0.024	0.10 ± 0.035	0.005
C17:1	0.17 ± 0.061	0.16 ± 0.058	0.635
C18:0	0.90 ± 0.307	1.01 ± 0.294	0.043
C18:1	1.30 ± 0.524	1.89 ± 0.855	<0.001
C18:2	0.68 ± 0.252	0.91 ± 0.348	0.001
C20:0	0.01 ± 0.003	0.01 ± 0.027	0.164
C20:1n9	0.05 ± 0.018	0.06 ± 0.028	0.054
C20:2	0.02 ± 0.023	0.03 ± 0.035	0.023
C20:3n6	0.24 ± 0.080	0.31 ± 0.157	0.029
C20:4n6	0.74 ± 0.229	0.65 ± 0.307	0.015
C20:5	0.44 ± 0.238	0.46 ± 0.175	0.977
C23:0	0.07 ± 0.019	0.08 ± 0.035	0.263
C24:0	0.17 ± 0.045	0.16 ± 0.040	0.085
C24:1	0.31 ± 0.045	0.28 ± 0.069	0.021
C22:6	0.40 ± 0.945	0.34 ± 0.169	0.019
Total	7.72 ± 2.247	9.29 ± 2.970	0.003

^1^ Mann–Whitney test.

**Table 4:** Total FAs in the blood of hyperlipidemic patients and normolipidemic controls following stratification according to *PNPLA3* I148M and *ABCB1* G2677T genotypes.

Genotype	Group		*p* ^1^
***PNPLA3* I148M**	**Normolipidemics (*n*)**	**Hyperlipidemics (*n*)**	
CC (II)	7.59 ± 2.463 (21)	9.44 ± 3.374 (26)	0.041
CG (IM)	8.08 ± 2.262 (15)	8.25 ± 1.946 (27)	0.795
GG (MM)	6.70 ± 1.421 (4)	12.57 ± 2.541 (8)	0.002
*p* ^2^	0.601	0.006	
***ABCB1* G2677T**			
GG	8.23 ± 2.524 (15)	8.42 ± 2.380 (26)	0.817
GT	7.92 ± 2.470 (16)	9.43 ± 2.577 (22)	0.078
TT	6.73 ± 1.044 (11)	10.78 ± 4.044 (14)	0.003
*p* ^2^	0.282	0.056	

^1^ Student *t*-test; ^2^ ANCOVA with body mass index (BMI), HbA1c as covariates.

**Table 5:** Effect of *PNPLA3* I148M (recessive model) on individual FA concentration according to multiple regression analysis.

FA	Adjusted R^2^		
	*PNPLA3* I148M (*p*)	+BMI (*p*)	+BMI + HbA1c (*p*)
C16:0	0.145 (0.001)	0.150 (0.259)	0.171 (0.118)
C18:0	0.262 (<0.001)	0.256 (0.470)	0.276 (0.108)
C18:1	0.175 (<0.001)	0.178 (0.277)	0.226 (0.038)
C18:2	0.132 (0.002)	0.164 (0.075)	0.151 (0.754)
C20:4	−0.011 (0.562)	−0.004 (0.422)	−0.013 (0.531)
C20:5	0.325 (<0.001)	0.313 (0.921)	0.304 (0.644)

## Data Availability

The data presented in this study are available within the article and supplementary material.

## Supplementary Materials

The following are available online at https://www.mdpi.com/2218-1989/11/2/90/s1, Figure S1. Comparison of total blood fatty acid concentrations in the three genotypes of the *PNPLA3* I148M (left) and the *ABCB1* G2677T (right) polymorphism, in normolipidemic controls and hyperlipidemic patients. Table S1. FA concentrations in individual samples. Table S2. 16:0 FA in the blood of hyperlipidemic patients and normoli-pidemic controls following stratification according to *PNPLA3* I148M and *ABCB1* G2677T genotypes. Table S3. 18:0 FA in the blood of hyperlipidemic patients and normolipidemic controls following stratification according to *PNPLA3* I148M and *ABCB1* G2677T genotypes. Table S4. 18:1 FA in the blood of hyperlipidemic patients and normolipidemic controls following stratification according to *PNPLA3* I148M and *ABCB1* G2677T genotypes. Table S5. 18:2 FA in the blood of hyperlipidemic patients and normolipidemic controls following stratification accord-ing to *PNPLA3* I148M and *ABCB1* G2677T genotypes. Table S6. 20:4 FA in the blood of hyperlipidemic patients and normolipidemic controls following stratification accord-ing to *PNPLA3* I148M and *ABCB1* G2677T genotypes. Table S7. 20:5 FA in the blood of hyperlipidemic patients and normolipidemic controls following stratification accord-ing to *PNPLA3* I148M and *ABCB1* G2677T genotypes. Table S8. Total cholesterol, triglycerides and glycated hemoglobin in the blood of hyperlipidemic patients and nor-molipidemic controls following stratification according to *PNPLA3* I148M and *ABCB1* G2677T genotypes.
