# Supplementary material for: A Study of Blood Fatty Acids Profile in Hyperlipidemic and Normolipidemic Subjects in Association with Common PNPLA3 and ABCB1 Polymorphisms
Source: Metabolites. 2021 Feb 4;11(2):90. doi: 10.3390/metabo11020090 (PMC7915980; doi:10.3390/metabo11020090)
Supplement: Supplementary file 1 [file metabolites-11-00090-s001.pdf]

SUPPLEMENTARY MATERIAL

Table S1. FA concentrations in individual samples.

CONTROL	C14:0	C15:0	C15:1	C16:0	C16:1	C17:0	C17:1	C18:0	C18:1cis	C18:2cis	C20:0	C20:1n9	C20:2	C20:3n6	C20:4n6	C20:5	C23:0	C24:0	C24:1	C22:6	SUM
**1**	0.12	0.02	0.09	1.53	0.22	0.03	0.40	0.72	1.46	0.53	0.01	0.02	0.07	0.11	0.39	0.15	0.03	0.05	0.10	0.06	6.11
**2**	0.03	0.01	0.05	0.58	0.05	0.02	0.22	0.29	0.50	0.31	0.01	0.01	0.07	0.07	0.43	0.10	0.02	0.03	0.05	0.05	2.89
**3**	0.01	0.01	0.03	0.50	0.03	0.01	0.15	0.26	0.32	0.17	0.01	0.01	0.06	0.03	0.40	0.06	0.02	0.02	0.03	0.02	2.14
**4**	0.02	0.01	0.05	0.62	0.04	0.01	0.19	0.34	0.46	0.26	0.01	0.01	0.06	0.07	0.40	0.12	0.03	0.04	0.07	0.03	2.83
**5**	0.06	0.01	0.06	0.53	0.02	0.02	0.31	0.33	0.39	0.34	0.01	0.01	0.06	0.07	0.40	0.08	0.03	0.03	0.08	0.10	2.95
**6**	0.03	0.01	0.06	0.89	0.07	0.02	0.25	0.44	0.60	0.34	0.01	0.01		0.09	0.32	0.18	0.04	0.07	0.09	0.04	3.55
**7**	0.05	0.01	0.06	0.72	0.05	0.02	0.30	0.36	0.60	0.37	0.01	0.01	0.07	0.07	0.36	0.11	0.03	0.05	0.08	0.04	3.34
**8**	0.05	0.01	0.04	0.68	0.07	0.02	0.19	0.36	0.65	0.26	0.01	0.02		0.10	0.44	0.12	0.03	0.05	0.07	0.13	3.24
**9**	0.04	0.02	0.06	0.86	0.11	0.02	0.25	0.42	0.72	0.34	0.01	0.01	0.06	0.12	0.46	0.13	0.04	0.05	0.08	0.05	3.86
**10**	0.04	0.01	0.05	0.63	0.03	0.02	0.22	0.38	0.36	0.28	0.01	0.01		0.06	0.45	0.12	0.03	0.05	0.05	0.03	2.81
**11**	0.07	0.01	0.10	0.61	0.04	0.01	0.41	0.35	0.36	0.32	0.01	0.01		0.08	0.39	0.12	0.03	0.05	0.08	0.08	3.13
**12**	0.09	0.01	0.05	0.72	0.04	0.02	0.24	0.33	0.56	0.36	0.01	0.01	0.07	0.09	0.46	0.13	0.04	0.04	0.06	0.05	3.37
**13**	0.10	0.02	0.06	0.89	0.05	0.02	0.31	0.52	0.95	0.45	0.01	0.02		0.11	0.38		0.04	0.04	0.08	0.05	4.09
**14**	0.07	0.01	0.06	1.15	0.13	0.02	0.23	0.58	0.77	0.29	0.01	0.02		0.08	0.32		0.02	0.04	0.07	0.07	3.94
**15**	0.15	0.01	0.08	0.88	0.08	0.01	0.30	0.54	0.51	0.28	0.02	0.02		0.10	0.36		0.03	0.05	0.07	0.04	3.51
**16**	0.04	0.01	0.02	0.61	0.07	0.01	0.11	0.26	0.61	0.16	0.01	0.01	0.07	0.06	0.40	0.06	0.01	0.03	0.04	0.02	2.62
**17**	0.01	0.01	0.06	0.59	0.03	0.02	0.33	0.33	0.41	0.34	0.01	0.01		0.08	0.41	0.12	0.03	0.05	0.07	0.04	2.94
**18**	0.04	0.01	0.05	0.49	0.04	0.01	0.22	0.25	0.34	0.22	0.01	0.01		0.08	0.41	0.10	0.03	0.04	0.05	0.03	2.41
**19**	0.04	0.01	0.06	0.79	0.06	0.02	0.27	0.43	0.62	0.34	0.01	0.01		0.09	0.43	0.12	0.03	0.05	0.07	0.08	3.53
**20**	0.02	0.01	0.05	0.57	0.04	0.02	0.27	0.35	0.55	0.35	0.01	0.01	0.06	0.11	0.43	0.11	0.04	0.04	0.07	0.08	3.18
**21**	0.07	0.02	0.09	0.85	0.06	0.03	0.36	0.47	0.76	0.67	0.01	0.01	0.06	0.15	0.42	0.16	0.05	0.05	0.07	0.10	4.47
**22**	0.07	0.01	0.05	0.62	0.06	0.02	0.26	0.30	0.63	0.31	0.01	0.01	0.07	0.10	0.42	0.06	0.01	0.02	0.05	0.05	3.15
**23**	0.08	0.01	0.03	0.41	0.03	0.02	0.13	0.19	0.38	0.13	0.01	0.01		0.06	0.41	0.06	0.02	0.03	0.05	0.02	2.08
**24**	0.07	0.01	0.07	0.65	0.04	0.02	0.30	0.39	0.40	0.51	0.01	0.01		0.11	0.42	0.12	0.03	0.04	0.05	0.04	3.31
**25**	0.06	0.01	0.02	0.33	0.01	0.01	0.12	0.19	0.35	0.16	0.00	0.00		0.03	0.38	0.05	0.02	0.02	0.04	0.02	1.83
**26**	0.04	0.01	0.07	0.57	0.05	0.01	0.32	0.32	0.40	0.19	0.01	0.01		0.06	0.46	0.09	0.02	0.04	0.06	0.07	2.78
**27**	0.05	0.01	0.06	0.82	0.04	0.02	0.32	0.55	0.57	0.62	0.01	0.01		0.13	0.41	0.12	0.02	0.05	0.07	0.05	3.93
**28**	0.03	0.01	0.08	0.51	0.04	0.01	0.40	0.35	0.45	0.30	0.01	0.01	0.07	0.11	0.52	0.13	0.03	0.04	0.08	0.06	3.26
**29**	0.14	0.02	0.04	0.87	0.02	0.02	0.16	0.53	0.47	0.33	0.01	0.01		0.11	0.35		0.03	0.04	0.05	0.04	3.24
**30**	0.09	0.03	0.10	1.25	0.08	0.03	0.30	0.76	0.84	0.65	0.01	0.01		0.20	0.46	0.04	0.03	0.05	0.05	0.09	5.07
**31**	0.10	0.02	0.06	1.08	0.09	0.03	0.28	0.72	0.74	0.31	0.01	0.01		0.13	0.38		0.02	0.05	0.07	0.04	4.12
**32**	0.06	0.01	0.06	0.59	0.06	0.01	0.28	0.31	0.33	0.26	0.01	0.01	0.06	0.07	0.44	0.11	0.01	0.04	0.06	0.05	2.81
**33**	0.04	0.01	0.05	0.71	0.04	0.02	0.22	0.37	0.53	0.24	0.01	0.01		0.07	0.41	0.12	0.03	0.05	0.06	0.04	3.04
**34**	0.03	0.01	0.04	0.42	0.02	0.02	0.18	0.27	0.32	0.25	0.01	0.01		0.07	0.38	0.07	0.02	0.02	0.04	0.04	2.22
**35**	0.02	0.01	0.07	0.61	0.04	0.02	0.35	0.39	0.45	0.37	0.01	0.01	0.07	0.11	0.37	0.11	0.02	0.04	0.07	0.03	3.17

**36**	0.03	0.01	0.04	0.62	0.08	0.01	0.17	0.31	0.41	0.26	0.01	0.01	0.07	0.10	0.34	0.10	0.03	0.03	0.05	0.03	2.71
**37**	0.05	0.01	0.03	0.74	0.09	0.01	0.15	0.42	0.68	0.23	0.01	0.01		0.09	0.31	0.10	0.03	0.04	0.06	0.03	3.09
**38**	0.03	0.01	0.04	0.62	0.05	0.01	0.16	0.32	0.33	0.25	0.01	0.01	0.06	0.07	0.39	0.11	0.03	0.04	0.06	0.02	2.63
**39**	0.03	0.01	0.07	0.64	0.04	0.01	0.34	0.35	0.47	0.29	0.01	0.01		0.09	0.36	0.11	0.03	0.04	0.08	0.07	3.03
**40**	0.03	0.01	0.07	0.61	0.04	0.01	0.31	0.33	0.45	0.28	0.01	0.01	0.06	0.09	0.44	0.11	0.02	0.04	0.07	0.05	3.04
**41**	0.03	0.01	0.10	0.76	0.04	0.02	0.47	0.55	0.56	0.39	0.01	0.01	0.06	0.12	0.41	0.14	0.03	0.05	0.08	0.09	3.95
**42**	0.04	0.01	0.08	0.63	0.03	0.02	0.43	0.39	0.49	0.36	0.01	0.01		0.09	0.48	0.13	0.04	0.04	0.08	0.08	3.43

HYPERLIPIDEMICS	C14:0	C15:0	C15:1	C16:0	C16:1	C17:0	C17:1	C18:0	C18:1cis	C18:2cis	C20:0	C20:1n9	C20:2	C20:3n6	C20:4n6	C20:5	C23:0	C24:0	C24:1	C22:6	SUM
**1**	0.09	0.02	0.07	0.94	0.06	0.02	0.34	0.49	0.62	0.36	0.01	0.01	0.06	0.12	0.39	0.10	0.02	0.04	0.07	0.08	**3.93**
**2**	0.11	0.02	0.08	1.03	0.06	0.03	0.37	0.62	0.83	0.41	0.01	0.01	0.07	0.11	0.44	0.13	0.03	0.05	0.09	0.10	**4.60**
**3**	0.12	0.02	0.05	1.22	0.11	0.03	0.25	0.56	0.96	0.49	0.01	0.01		0.12	0.38	0.12	0.04	0.04	0.05	0.06	**4.64**
**4**	0.07	0.01	0.03	0.55	0.03	0.01	0.14	0.30	0.52	0.35	0.01	0.02		0.08	0.34	0.06	0.03	0.02	0.03	0.03	**2.62**
**5**	0.07	0.01	0.08	1.07	0.04	0.02	0.42	0.64	1.10	0.61	0.01	0.01	0.06	0.13	0.42	0.12	0.04	0.04	0.07	0.18	**5.15**
**6**	0.08	0.01	0.05	1.25	0.09	0.02	0.25	0.48	0.97	0.39	0.02	0.01	0.06	0.06	0.33	0.11	0.02	0.03	0.05	0.04	**4.33**
**7**	0.11	0.02	0.08	1.36	0.11	0.02	0.36	0.52	1.02	0.53	0.01	0.02	0.06	0.10	0.41	0.07	0.01	0.03	0.06	0.08	**5.00**
**8**	0.17	0.03	0.06	1.20	0.11	0.03	0.31	0.56	0.88	0.43	0.01	0.01	0.07	0.08	0.35	0.13	0.02	0.05	0.07	0.02	**4.57**
**9**	0.09	0.03	0.06	1.34	0.11	0.03	0.29	0.59	1.18	0.59	0.01	0.02	0.07	0.23	0.48	0.13	0.05	0.04	0.06	0.06	**5.46**
**10**	0.15	0.01	0.01	0.93	0.05	0.02	0.25	0.51	0.60	0.45	0.01	0.01	0.07	0.11	0.37	0.11	0.02	0.03	0.05	0.07	**3.86**
**11**	0.09	0.01	0.08	0.78	0.05	0.01	0.39	0.46	0.63	0.59	0.01	0.01	0.06	0.14	0.43	0.10	0.03	0.04	0.06	0.13	**4.11**
**12**	0.10	0.02	0.07	1.07	0.09	0.02	0.29	0.51	0.77	0.56	0.01	0.01	0.07	0.14	0.40	0.12	0.04	0.04	0.05	0.07	**4.43**
**13**	0.13	0.02	0.05	1.21	0.13	0.03	0.25	0.61	1.35	0.37	0.01	0.02	0.07	0.12	0.37	0.10	0.04	0.03	0.06	0.03	**5.01**
**14**	0.09	0.01	0.05	0.68	0.03	0.02	0.24	0.38	0.38	0.31	0.01	0.01	0.07	0.07	0.34	0.11	0.03	0.03	0.04	0.03	**2.92**
**15**	0.12	0.01	0.04	0.57	0.04	0.01	0.15	0.34	0.53	0.28	0.01	0.01	0.06	0.08	0.32	0.09	0.03	0.03	0.06	0.08	**2.86**
**16**	0.09	0.01	0.04	0.86	0.06	0.02	0.19	0.46	0.71	0.16	0.01	0.01	0.06	0.06	0.33	0.09	0.02	0.04	0.07	0.03	**3.34**
**17**	0.06	0.01	0.04	0.77	0.07	0.01	0.18	0.36	0.64	0.33	0.01	0.01		0.12	0.31	0.07	0.02	0.03	0.05	0.05	**3.15**
**18**	0.09	0.01	0.05	0.60	0.03	0.02	0.20	0.34	0.47	0.25	0.01	0.01	0.06	0.06	0.32	0.12	0.04	0.04	0.06	0.03	**2.82**
**19**	0.05	0.00	0.04	0.40	0.05	0.01	0.17	0.25	0.38	0.16	0.00	0.01		0.05	0.33	0.06	0.02	0.03	0.04	0.04	**2.09**
**20**	0.10	0.01	0.04	0.63	0.11	0.01	0.22	0.31	0.60	0.28	0.01	0.01	0.06	0.15	0.34	0.07	0.02	0.03	0.04	0.08	**3.13**
**21**	0.10	0.01	0.03	0.73	0.05	0.01	0.16	0.29	0.64	0.31	0.01	0.01	0.07	0.05	0.31	0.08	0.02	0.03	0.04	0.04	**3.00**
**22**	0.15	0.01	0.05	0.76	0.05	0.01	0.23	0.38	0.63	0.34	0.01	0.01	0.06	0.06	0.35	0.10	0.02	0.04	0.06	0.05	**3.40**
**23**	0.12	0.02	0.06	0.93	0.08	0.03	0.28	0.46	0.65	0.64	0.01	0.01	0.06	0.14	0.40	0.10	0.03	0.03	0.04	0.11	**4.19**
**24**	0.10	0.01	0.03	0.55	0.07	0.01	0.12	0.27	0.39	0.18	0.01	0.01	0.07	0.08	0.29	0.07	0.02	0.03	0.03	0.03	**2.36**
**25**	0.07	0.01	0.03	0.56	0.05	0.02	0.15	0.29	0.47	0.20	0.01	0.01	0.06	0.05	0.31	0.07	0.02	0.03	0.03	0.06	**2.49**
**26**	0.09	0.01	0.05	0.65	0.05	0.01	0.25	0.36	0.55	0.25	0.01	0.01	0.06	0.05	0.32	0.07	0.02	0.03	0.05	0.03	**2.94**
**27**	0.08	0.03	0.08	1.20	0.20	0.02	0.33	0.67	1.01	0.74	0.01	0.02	0.06	0.32	0.51	0.04	0.05	0.04	0.05	0.08	**5.53**
**28**	0.08	0.01	0.05	1.01	0.09	0.02	0.23	0.52	1.01	0.50	0.01	0.01	0.06	0.13	0.39	0.02	0.04	0.05	0.08	0.07	**4.40**
**29**	0.05	0.01	0.05	0.73	0.07	0.01	0.30	0.38	0.76	0.45	0.01	0.01		0.10	0.35	0.09	0.02	0.03	0.04	0.05	**3.52**
**30**	0.09	0.02	0.08	1.02	0.11	0.03	0.37	0.54	0.68	0.63	0.01	0.01	0.07	0.12	0.41	0.10	0.04	0.04	0.05	0.09	**4.50**

**31**	0.08	0.02	0.07	1.13	0.08	0.03	0.35	0.46	1.09	0.60	0.01	0.01	0.07	0.09	0.43	0.11	0.04	0.04	0.07	0.10	**4.88**
**32**	0.06	0.01	0.04	0.61	0.05	0.02	0.20	0.32	0.43	0.29	0.01	0.01	0.07	0.08	0.35	0.09	0.03	0.04	0.05	0.05	**2.79**
**33**	0.08	0.02	0.04	0.79	0.10	0.02	0.22	0.37	0.80	0.43	0.01	0.01	0.06	0.11	0.39	0.08	0.02	0.03	0.05	0.08	**3.71**
**34**	0.08	0.01	0.05	0.92	0.07	0.02	0.26	0.41	0.76	0.28	0.01	0.01	0.06	0.08	0.34	0.13	0.03	0.05	0.06	0.03	**3.67**
**35**	0.09	0.01	0.07	0.75	0.09	0.02	0.32	0.42	0.70	0.55	0.01	0.01	0.06	0.14	0.40	0.07	0.02	0.03	0.05	0.09	**3.90**
**36**	0.08	0.01	0.07	0.94	0.08	0.02	0.31	0.45	0.79	0.41	0.01	0.01		0.11	0.39	0.12	0.02	0.05	0.06	0.09	**4.02**
**37**	0.09	0.01	0.04	0.89	0.06	0.02	0.20	0.38	0.78	0.29	0.01	0.01	0.07	0.06	0.31	0.10	0.02	0.04	0.05	0.02	**3.45**
**38**	0.08	0.01	0.04	0.75	0.08	0.02	0.14	0.35	0.56	0.37	0.01	0.01	0.06	0.09	0.33	0.12	0.03	0.04	0.05	0.05	**3.18**
**39**	0.06	0.02	0.06	0.89	0.06	0.02	0.26	0.42	0.71	0.38	0.01	0.01	0.06	0.08	0.39	0.14	0.04	0.05	0.08	0.06	**3.80**
**40**	0.14	0.02	0.09	1.01	0.10	0.02	0.37	0.46	0.73	0.57	0.01	0.01		0.15	0.40	0.12	0.03	0.05	0.06	0.09	**4.43**
**41**	0.06	0.01	0.06	0.77	0.06	0.02	0.33	0.37	0.70	0.33	0.01	0.01	0.07	0.12	0.38	0.09	0.02	0.05	0.07	0.07	**3.60**
**42**	0.11	0.01	0.05	0.82	0.04	0.02	0.34	0.37	0.64	0.49	0.01	0.01	0.06	0.06	0.44	0.09	0.03	0.04	0.04	0.07	**3.73**
**43**	0.14	0.02	0.05	1.33	0.17	0.03	0.24	0.56	1.02	0.47	0.01	0.01	0.07	0.17	0.37	0.16	0.04	0.05	0.07	0.05	**5.02**
**44**	0.07	0.01	0.09	0.82	0.06	0.02	0.41	0.49	0.73	0.45	0.01	0.01	0.07	0.11	0.43	0.11	0.03	0.05	0.06	0.07	**4.09**
**45**	0.06	0.01	0.04	0.66	0.05	0.02	0.18	0.37	0.50	0.32	0.01	0.01	0.07	0.10	0.37	0.09	0.03	0.04	0.06	0.06	**3.03**
**46**	0.05	0.01	0.05	0.80	0.05	0.01	0.23	0.31	0.59	0.31	0.01	0.01	0.06	0.05	0.35	0.09	0.03	0.05	0.08	0.08	**3.23**
**47**	0.12	0.03	0.08	1.15	0.11	0.03	0.30	0.54	1.20	0.69	0.01	0.01		0.22	0.43	0.11	0.04	0.04	0.07	0.18	**5.35**
**48**	0.05	0.01	0.07	0.91	0.08	0.01	0.32	0.46	0.79	0.38	0.01	0.01		0.13	0.39	0.03	0.03	0.04	0.05	0.14	**3.93**
**49**	0.09	0.03	0.07	1.92	0.19	0.04	0.32	0.80	2.61	0.54	0.02	0.04		0.17	0.39	0.02	0.07	0.05	0.09	0.08	**7.52**
**50**	0.12	0.03	0.09	1.71	0.18	0.04	0.41	0.80	1.48	0.69	0.01	0.03		0.35	0.35	0.02	0.04	0.05	0.07	0.06	**6.53**
**51**	0.09	0.02	0.05	1.37	0.10	0.03	0.31	0.70	1.24	0.62	0.01	0.02		0.15	0.36	0.02	0.04	0.04	0.07	0.10	**5.36**
**52**	0.16	0.02	0.06	1.65	0.26	0.03	0.30	0.64	1.66	0.76	0.01	0.03	0.06	0.12	0.36	0.11	0.03	0.06	0.08	0.07	**6.47**
**53**	0.21	0.04	0.06	1.97	0.43	0.05	0.26	0.76	1.99	0.66	0.02	0.02	0.08	0.23	0.39	0.18	0.06	0.07	0.07	0.15	**7.69**
**54**	0.10	0.02	0.06	0.96	0.14	0.02	0.24	0.45	0.90	0.68	0.03	0.01	0.06	0.19	0.59	0.11	0.04	0.04	0.06	0.08	**4.80**
**55**	0.09	0.02	0.08	1.11	0.10	0.02	0.28	0.47	0.97	0.65	0.01	0.01	0.11	0.19	0.39	0.16	0.05	0.05	0.07	0.10	**4.93**
**56**	0.11	0.02	0.05	0.92	0.08	0.02	0.21	0.41	0.80	0.53	0.01	0.01	0.07	0.13	0.37	0.13	0.04	0.04	0.05	0.06	**4.04**
**57**	0.09	0.02	0.09	1.03	0.10	0.02	0.38	0.54	1.04	0.76	0.01	0.02	0.06	0.17	0.65	0.12	0.05	0.05	0.07	0.10	**5.38**
**58**	0.19	0.03	0.09	2.24	0.34	0.03	0.46	0.72	1.95	0.74	0.01	0.02	0.07	0.19	0.45	0.16	0.04	0.06	0.09	0.12	**8.01**
**59**	0.08	0.02	0.08	0.81	0.08	0.02	0.25	0.34	0.58	0.46	0.01	0.01	0.07	0.08	0.36	0.10	0.03	0.03	0.06	0.05	**3.52**
**60**	0.15	0.03	0.06	1.23	0.10	0.03	0.25	0.45	1.15	0.41	0.01	0.01	0.06	0.10	0.32	0.13	0.04	0.05	0.06	0.07	**4.72**
**61**	0.05	0.01	0.03	0.48	0.04	0.01	0.13	0.25	0.43	0.19	0.01	0.01	0.07	0.07	0.44	0.06	0.02	0.02	0.03	0.03	**2.37**
**62**	0.06	0.01	0.03	0.85	0.14	0.01	0.16	0.35	0.73	0.20	0.01	0.01		0.09	0.36	0.07	0.01	0.03	0.05	0.03	**3.19**

Table S2. 16:0 FA in the blood of hyperlipidemic patients and normolipidemic controls following stratification according to PNPLA3 I148M and ABCB1 G2677T genotypes.

**Genotype**	**Group**		
***PNPLA3* I148M**	**Normolipidemics (*n*)**	**Hyperlipidemics (*n*)**	p^1
CC	1.45 ± 0.595 (21)	1.84 ± 0.781 (26)	0.053
CG	1.33 ± 0.426 (15)	1.60 ± 0.401 (27)	0.052
GG	1.13 ± 0.290 (4)	2.52 ± 0.705 (8)	0.001
p^2	0.564	0.016	
***ABCB1* G2677T**			
GG	1.47 ± 0.506 (15)	1.64 ± 0.522 (26)	0.297
GT	1.40 ± 0.598 (16)	1.85 ± 0.626 (22)	0.033
TT	1.20 ± 0.186 (11)	2.09 ± 0.939 (14)	0.004
p^2	0.485	0.071	

[1] Student t-test; [2] ANCOVA with BMI, HbA1c as covariates.

Table S3. 18:0 FA in the blood of hyperlipidemic patients and normolipidemic controls following stratification according to PNPLA3 I148M and ABCB1 G2677T genotypes.

**Genotype**	**Group**		
***PNPLA3* I148M**	**Normolipidemics (*n*)**	**Hyperlipidemics (*n*)**	p^1
CC	0.94 ± 0.339 (21)	1.01 ± 0.297 (26)	0.457
CG	0.88 ± 0.291 (15)	0.89 ± 0.185 (27)	0.960
GG	0.72 ± 0.213 (4)	1.40 ± 0.277 (8)	0.002
p^2	0.488	<0.001	
***ABCB1* G2677T**			
GG	0.91 ± 0.273 (15)	0.98 ± 0.276 (26)	0.417
GT	0.94 ± 0.399 (16)	1.02 ± 0.309 (22)	0.492
TT	0.83 ± 0.188 (11)	1.02 ± 0.326 (14)	0.109
p^2	0.749	0.776	

[1] Student t-test; [2] ANCOVA with BMI, HbA1c as covariates.

Table S4. 18:1 FA in the blood of hyperlipidemic patients and normolipidemic controls following stratification according to PNPLA3 I148M and ABCB1 G2677T genotypes.

**Genotype**	**Group**		
***PNPLA3* I148M**	**Normolipidemics (*n*)**	**Hyperlipidemics (*n*)**	p^1
CC	1.33 ± 0.660 (21)	1.87 ± 0.831 (26)	0.019
CG	1.27 ± 0.326 (15)	1.65 ± 0.515 (27)	0.015
GG	1.08 ± 0.244 (4)	1.40 ± 0.277 (8)	0.023
p^2	0.707	0.009	
***ABCB1* G2677T**			
GG	1.41 ± 0.602 (15)	1.65 ± 0.551 (26)	0.210
GT	1.37 ± 0.552 (16)	1.98 ± 0.961 (22)	0.030
TT	1.05 ± 0.171 (11)	2.24 ± 1.064 (14)	0.001
p^2	0.190	0.030	

[1] Student t-test; [2] ANCOVA with BMI, HbA1c as covariates.

Table S5. 18:2 FA in the blood of hyperlipidemic patients and normolipidemic controls following stratification according to PNPLA3 I148M and ABCB1 G2677T genotypes.

**Genotype**	**Group**		
***PNPLA3* I148M**	**Normolipidemics (*n*)**	**Hyperlipidemics (*n*)**	p^1
CC	0.64 ± 0.204 (21)	0.91 ± 0.382 (26)	0.004
CG	0.76 ± 0.315 (15)	0.82 ± 0.281 (27)	0.533
GG	0.55 ± 0.198 (4)	1.26 ± 0.268 (8)	0.001
p^2	0.233	0.028	
***ABCB1* G2677T**			
GG	0.67 ± 0.173 (15)	0.081 ± 0.335 (26)	0.091
GT	0.76 ± 0.350 (16)	0.95 ± 0.281 (22)	0.064
TT	0.59 ± 0.125 (11)	1.03 ± 0.438 (14)	0.002
p^2	0.249	0.200	

[1] Student t-test; [2] ANCOVA with BMI, HbA1c as covariates.

Table S6. 20:4 FA in the blood of hyperlipidemic patients and normolipidemic controls following stratification according to PNPLA3 I148M and ABCB1 G2677T genotypes.

**Genotype**	**Group**		
***PNPLA3* I148M**	**Normolipidemics (*n*)**	**Hyperlipidemics (*n*)**	***p*[1]**
CC	0.67 ± 0.213 (21)	0.70 ± 0.331 (26)	0.767
CG	0.80 ± 0.255 (15)	0.59 ± 0.303 (27)	0.030
GG	0.76 ± 0.087 (4)	0.71 ± 0.250 (8)	0.693
p[2]	0.289	0.239	
***ABCB1* G2677T**			
GG	0.71 ± 0.221 (15)	0.58 ± 0.271 (26)	0.117
GT	0.82 ± 0.197 (16)	0.63 ± 0.170 (22)	0.003
TT	0.64 ± 0.256 (11)	0.82 ± 0.459 (14)	0.259
p[2]	0.068	0.085	

[1] Student t-test; [2] ANCOVA with BMI, HbA1c as covariates.

Table S7. 20:5 FA in the blood of hyperlipidemic patients and normolipidemic controls following stratification according to PNPLA3 I148M and ABCB1 G2677T genotypes.

**Genotype**	**Group**		
***PNPLA3* I148M**	**Normolipidemics (*n*)**	**Hyperlipidemics (*n*)**	***p*[1]**
CC	0.40 ± 0.271 (21)	0.54 ± 0.152 (26)	0.048
CG	0.48 ± 0.190 (15)	0.48 ± 0.106 (27)	0.912
GG	0.50 ± 0.137 (5)	0.22 ± 0.184 (8)	0.023
p[2]	0.510	< 0.001	
***ABCB1* G2677T**			
GG	0.45 ± 0.266 (15)	0.44 ± 0.138 (26)	0.821
GT	0.42 ± 0.229 (16)	0.43 ± 0.183 (22)	0.866
TT	0.43 ± 0.231 (11)	0.59 ± 0.155 (14)	0.056
p[2]	0.936	0.005	

[1] Student t-test; [2] ANCOVA with BMI, HbA1c as covariates.

Table S8. Total cholesterol, triglycerides and glycated hemoglobin in the blood of hyperlipidemic patients and nor-molipidemic controls following stratification according to PNPLA3 I148M and ABCB1 G2677T genotypes.

	**Normolipidemics (n)**		**Hyperlipidemics (n)**				
***PNPLA3* I148M**	**TC (mmol/L)**	**TG (mmol/L)**	**TC (mmol/L)**	**TG (mmol/L)**	**HbA1c (%)**	***p*[1]**	***p*[2]**
CC	4.45 ± 0.677 (21)	1.13 ± 0.478	6.71 ± 1.015 (26)	2.46 ± 0.156	6.06 ± 1.126	< 0.001	< 0.001
CG	5.13 ± 0.441 (15)	1.08 ± 0.381	6.28 ± 0.938 (27)	2.25 ± 0.856	5.37 ± 0.639	< 0.001	< 0.001
GG	4.89 ± 1.200 (5)	1.47 ± 0.504	6.94 ± 0.830 (8)	2.60 ± 1.311	5.76 ± 0.612	0.010	0.192
p[3]	0.011	0.352	0.131	0.721	0.023		
ABCB1 G2677T							
GG	4.65 ± 0.831 (15)	1.15 ± 0.393	6.29 ± 1.072 (26)	2.29 ± 0.743	5.97 ± 1.012	< 0.001	< 0.001
GT	4.74 ± 0.711 (16)	1.18 ± 0.546	6.50 ± 0.806 (22)	2.50 ± 0.869	5.69 ± 0.831	< 0.001	< 0.001
TT	4.93 ± 0.441 (11)	1.05 ± 0.336	7.15 ± 0.799 (14)	2.41 ± 1.651	5.37 ± 0.843	< 0.001	0.009
p[3]	0.405	0.841	0.022	0.784	0.153		

[1] Student t-test, TC normolipidemics vs. hyperlipidemics; [2] Student t-test, TG normolipidemics vs. hyperlipidemics; [3] ANCOVA with BMI, HbA1c as covariates.

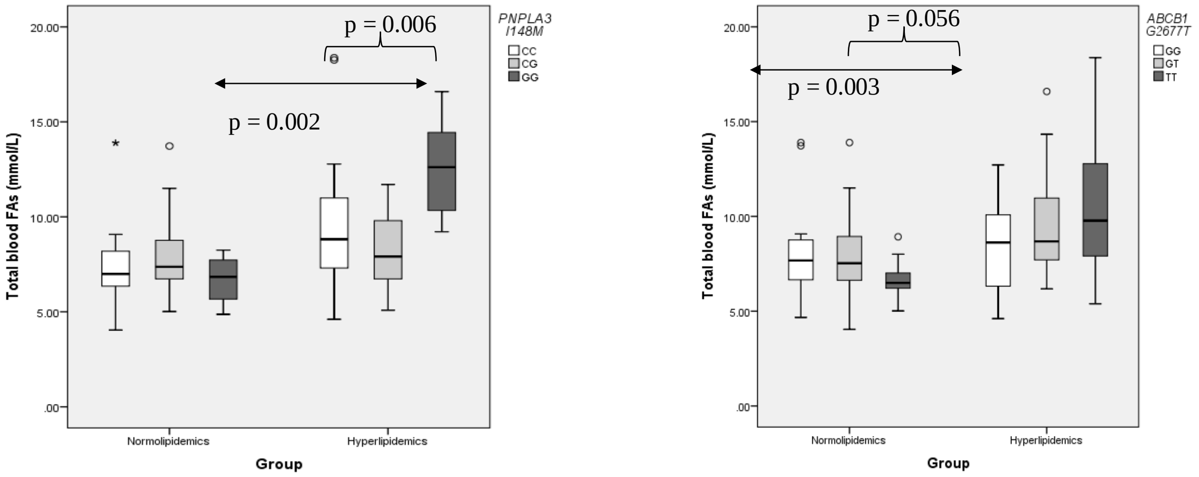

Figure S1. Comparison of total blood fatty acid concentrations in the three genotypes of the *PNPLA3* I148M (left) and the ABCB1 G2677T (right) polymorphism, in normolipidemic controls and hyperlipidemic patients .
